# High-protein feed elevates ruminal and plasma ammonia and is associated with altered open-field behavior in Suffolk sheep

**DOI:** 10.3389/fvets.2026.1808950

**Published:** 2026-05-22

**Authors:** Qianrige, Sanggun Roh, Michiru Fukasawa, Shin-ichiro Ogura

**Affiliations:** 1 Laboratory of Grassland-Animal Production and Ecology, Graduate School of Agriculture Science, Tohoku University, Osaki, Japan; 2 Laboratory of Animal Physiology Science, Graduate School of Agriculture Science, Tohoku University, Sendai, Japan

**Keywords:** open field test, rumen ammonia, rumen volatile fatty acid, rumen–brain axis, sheep, stress response

## Abstract

Rumen fermentation shapes metabolic and endocrine functions. However, its association with stress behavior in ruminants remains underexplored. Therefore, we evaluated the effects of feed energy and protein levels in physiological and behavioral stress responses by altering rumen production in Suffolk sheep. Seventeen Suffolk sheep were fed high-energy (HE), high-protein (HP), or control feeds. Behavior in the open field test (OFT) was quantified as the latency to enter the field, number of squares entered, frequency of bleats, sniffing, and latency to bleat. Moreover, rumen volatile fatty acid, ammonia, plasma ammonia, cortisol, and aldosterone levels were measured. The HE group was characterized by a lower acetate-to-propionate (A/P) ratio than that in the control. Similarly, HP sheep were characterized by high ruminal and plasma ammonia levels. In the OFT, both the HE and HP groups bleated and sniffed more frequently than the control group; the number of squares entered was greater in the HP group than in the control group. Plasma cortisol and aldosterone levels did not differ according to the feed. Furthermore, plasma ammonia levels correlated with a greater number of squares entered (*ρ* = 0.78), whereas a lower A/P ratio correlated with lower cortisol levels (*ρ* = −0.56). Our findings suggest that feed composition can modulate stress responses, particularly stress-related behaviors, in sheep. This study provides preliminary evidence that feed-induced changes in rumen metabolites, particularly ammonia, are associated with behavioral stress responses in sheep. These findings may help inform feeding strategies that consider animal welfare.

## 1 Introduction

Ruminants exhibit measurable behavioral and physiological responses during novelty- or stress-related tests. Behavioral indicators include increased vocalizations, greater locomotion, reduced latency to enter the test pen or bleat, heightened vigilance, and more escape attempts during open-field or novel-object tests (1–3). Physiologically, stress activates the sympathetic-adrenal-medullary and hypothalamic–pituitary–adrenal axes. This leads to catecholamine and cortisol secretion and accompanying changes in the heart/respiration rate and energy metabolism (4–6).

These behavioral and physiological responses may also be modulated by ruminal metabolites. In monogastric animals, microbially derived volatile fatty acids (VFAs; acetate, propionate, and butyrate) signal to the brain via the gut–brain axis to modulate neurophysiology and behavior (7). Rumen VFAs can engage cellular stress signaling. For example, butyrate exposure is associated with transient adaptive stress responses in the rumen epithelium (8). In our previous study, a high-energy (HE) feed increased escape attempts (9). Additionally, the ruminal acetate-to-propionate (A/P) ratio was positively associated with the number of contacts with a novel object, an index of curiosity during the arena test (9). These findings suggest that ruminal fermentation patterns alter behavioral stress responsiveness in ruminants.

The contribution of ruminal ammonia to behavioral stress responses remains largely understudied. Ammonia originates from the degradation of rumen-degradable protein. Most of it is utilized for microbial protein, and excess is absorbed into the blood (10). Elevated blood ammonia is neurotoxic and can affect brain function (11). It has also been linked to altered affective states in humans (12) and functional disturbances in ruminants. For example, parotid secretion was suppressed when jugular ammonia increased to approximately 4.8 mg/dL in sheep (13). Similarly, the risk of toxicity increases when ruminal ammonia exceeds approximately 140 mg/dL under high non-protein nitrogen intake (14). Ammonia can cross the blood–brain barrier and alter neurotransmission. Chronic moderate hyperammonemia impairs avoidance behavior and conditional discrimination learning in rats, demonstrating the measurable neurobehavioral effects of elevated blood ammonia levels (15). This suggests that higher circulating ammonia levels can modulate stress-related behaviors in ruminants.

In this study, we hypothesized that feed energy and protein levels modify behavioral and physiological stress responses in ruminants by altering rumen fermentation, particularly that of ammonia. This study may contribute to understanding how feed-induced rumen metabolic changes relate to behavioral stress responses in sheep.

## 2 Materials and methods

### 2.1 Ethics statement

The animal study protocol was approved by the Animal Care Committee of the Tohoku University (2019AgA-025).

### 2.2 Animals and treatments

This study was conducted in 2019 at the Kawatabi Field Science Center, Graduate School of Agricultural Science, Tohoku University (Osaki, Miyagi, Japan). Seventeen Suffolk sheep (*Ovis aries*) were included in this study. The sheep were housed individually in adjacent pens measuring 1.8 × 1.5 m with straw bedding. The pens were separated by horizontal wooden boards, allowing visual and auditory contact between animals. This arrangement was used during the experimental period to enable individual feeding management and health monitoring. They were divided into three feed groups to ensure a balance in sex, age in months, and body weight distribution. No significant differences were found among the three treatment groups in body weight (*p* = 0.998), age (*p* = 0.856; Kruskal–Wallis test), or sex ratio (*p* = 1.000; Fisher’s exact test), confirming that the groups were balanced at baseline (Table 1). The HE (*n* = 5) and control (*n* = 6) groups were fed 60:40 and 20:80 dry matter ratios of corn to Bermuda grass hay, respectively. In contrast, the high-protein (HP) group (*n* = 6) was fed a 23:77 dry matter ratio of soybean meal to Bermuda grass hay. The sheep were fed the same amount of dry matter (2.4% of their body weight) once daily at 10:30 a.m. Orts were collected and weighed before each feeding; they remained below 5% of the offered amount for all animals throughout the study. Water and mineral mixtures were provided ad libitum. The chemical composition of the experimental feed is outlined in Table 2.

**Table 1:** Treatment, period, weight, age, and sex of experimental sheep.

Treatment	Period	Sex	Age in months	Weight
HE	1	Female	11	49.5
HE	1	Male	11	41.5
HE	1	Female	11	38.0
HE	2	Male	51	80.5
HE	2	Male	28	77.0
HE (mean ± SD)			22.4 ± 17.6	57.3 ± 20.1
HP	1	Female	11	42.0
HP	1	Male	11	40.0
HP	1	Female	11	42.5
HP	2	Female	63	75.0
HP	2	Male	38	86.0
HP (mean ± SD)			27.0 ± 20.9	59.3 ± 20.1
Control	1	Female	11	38.5
Control	1	Male	11	44.5
Control	1	Female	11	43.5
Control	2	Female	63	76.0
Control	2	Male	39	69.0
Control	2	Male	38	77.0
Control (mean ± SD)			28.8 ± 21.5	58.1 ± 17.8

HE = high-energy feed (60:40 corn to Bermuda grass hay, DM basis); HP = high-protein feed (23:77 soybean meal to Bermuda grass hay); Control = 20:80 corn to Bermuda grass hay. Periods 1 and 2 refer to two separate experimental periods conducted due to space limitations at the facility. No significant differences among groups (body weight: *p* = 0.998; age: *p* = 0.856, Kruskal–Wallis test; sex ratio: *p* = 1.000, Fisher’s exact test).

**Table 2:** Chemical composition and nutritive value of three experimental feeds in the high-energy (HE), high-protein (HP), and control groups.

Item	HE	HP	Control
Ingredient (g/kg of DM)			
Corn	600	0	200
Soybean meal	0	230	0
Bermuda grass	400	770	800
Chemical composition (g/100 g of DM)			
Crude protein	9.0	18.9	9.5
Total digestible nutrient	77.9	63.1	63.3
Neutral detergent fiber	34	55	56
Acid detergent fiber	15	27	27
Ash	4.6	10.5	9.2

DM, dry matter.

### 2.3 Experimental design

**Figure 1:**
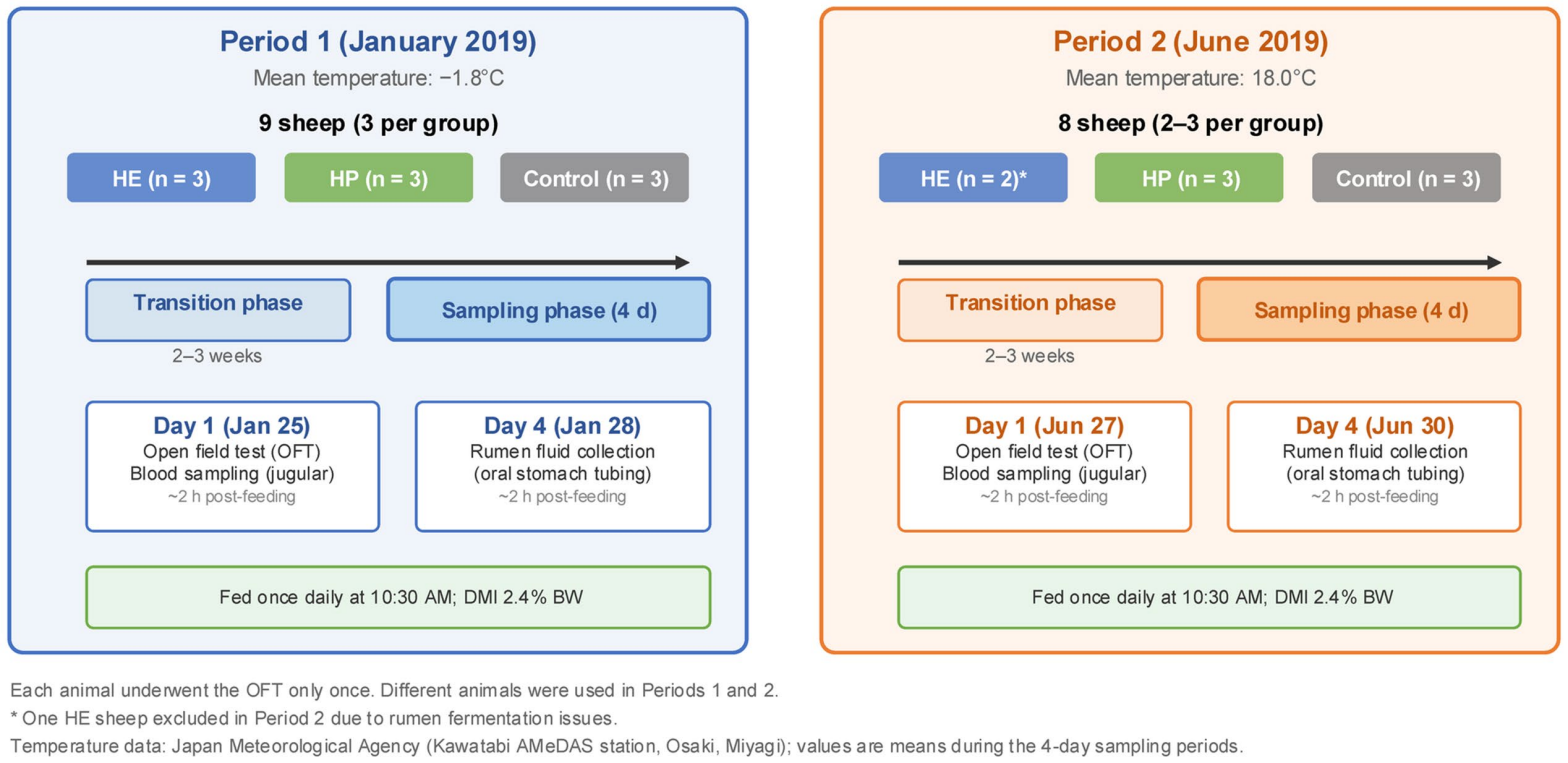
Schematic overview of the experimental design showing the timeline, group allocation, and sampling schedule for Periods 1 (January 25–28, 2019) and 2 (June 27–30, 2019). Mean ambient temperatures during the sampling periods were −1.8 °C and 18.0 °C for Periods 1 and 2, respectively (Japan Meteorological Agency, Kawatabi AMeDAS station, Osaki, Miyagi).

The experimental procedure was divided into two periods due to space limitations at the facility (Period 1: January 25–28, 2019; Period 2: June 27–30, 2019). In Periods 1 and 2, each of the three treatment groups was represented by three sheep; different animals were used in Periods 1 and 2, so each sheep underwent the OFT only once. One sheep in the HE group was excluded during Period 2 because of rumen fermentation issues, which reduced the group size to five. The experimental period was divided into two phases: transition and sampling phases lasting 2–3 weeks and 4 d, respectively. On day 1 of each sampling phase, an open field test (OFT) was conducted to observe the behavioral stress response to a novel environment. After completing the test for each animal, 10 mL of blood was collected via jugular venipuncture; restraint did not exceed 5 min per animal. On day 4 of each sampling period, 100 mL of ruminal fluid was collected from each animal using oral stomach tubing, 2 h after feeding. Rumen fluid sampling was conducted on a separate day from the OFT to avoid the additional stress associated with oral stomach tubing, which could have confounded the behavioral observations. The timing of rumen fluid sampling was selected to coincide with the OFT to maintain consistency; both procedures were conducted approximately 2 h after feeding. A schematic overview of the experimental design is shown in Figure 1.

### 2.4 OFT

Each sheep was placed in a start box (1.2 × 1.6 × 1.0 m) for 1 min to standardize pre-test conditions. The OFT arena (4 × 4 m) was enclosed by 1.8-m-high wooden panels that visually blocked the external environment on all sides. The start box door was then opened, and behaviors were recorded for 4 min from the moment the sheep entered the arena. Latency to enter was measured from the opening of the start box door until both hind legs crossed into the open field. If a sheep did not voluntarily leave the start box within 1 min, gentle intervention was planned; however, all sheep entered within this period. A grid of sixteen 1-m squares was marked on the floor with a rope to quantify locomotion. Two video cameras were mounted on wall tops at diagonal corners, ensuring no blind areas.

The behavioral variables observed during the OFT included latency to enter the field, number of squares entered, frequency of bleats, sniffing, and latency to bleat. Definitions of the behavioral variables observed during the OFT are provided in Table 3. All behavioral variables were scored from video recordings by a single observer.

**Table 3:** Behavioral variables observed during the 4-min open field test.

Behavior	Description
Latency to enter (s)	Time taken for a sheep to enter the test pen with both hind legs.
Squares entered (frequency)	Frequency of all four hooves passing over a marked grid line.
Sniffing (frequency)	Frequency of nose contact with the arena floor or wall.
Bleats (frequency)	Number of clearly audible vocalizations emitted during the test.
Latency to bleat (s)	Latency from test pen entry to the first bleat.

### 2.5 Measurement of stress-related hormones in the blood

Blood samples were centrifuged at 2,000 × *g* for 15 min at 4 °C, and the resulting plasma was collected and stored at −80 °C until hormone levels were measured. Plasma cortisol concentration was measured using the DetectX® Cortisol Enzyme Immunoassay Kit (K003-H1/H5; Arbor Assays, Ann Arbor, MI, USA). Plasma aldosterone levels were measured using an Aldosterone Chemiluminescent Immunoassay Kit (K052-C1; Arbor Assays).

### 2.6 Analysis of rumen fluid

The collected rumen fluid samples were filtered through double-layered medical gauze and stored in an icebox until centrifugation. After all the samples were collected, they were centrifuged at 10,000 × *g* for 4 min to remove solid particles. The samples were then stored at −80 °C until further analysis. The concentrations of VFAs (acetic acid, propionic acid, and butyric acid) in rumen fluid samples were measured using high performance liquid chromatography (Tohoku Electronic Industrial, Sendai, Japan). For each run, 20 μL of each sample was injected twice onto a Bio-Rad HPX 87H ion-exchange column (Bio-Rad Laboratories Ltd., Watford, UK), with dimensions of 300 mm by 7.8 mm, safeguarded by a 30 mm cation H + ion-exchange resin guard column. The mobile phase consisted of 0.005 M sulfuric acid, with the system maintained at a temperature of 50 °C and a flow rate set at 0.8 mL per minute. Rumen and plasma ammonia concentrations were measured using a colorimetric ammonia assay kit based on the Berthelot reaction (MET-5086; Cell Biolabs, San Diego, CA, USA).

### 2.7 Statistical analysis

Different statistical models were applied based on data characteristics to analyze the behavioral variables in the OFT. The frequencies of squares entered, sniffing of the ground or wall, and bleats were analyzed using a negative binomial regression with a log-link function, as these data showed overdispersion. The model included the treatment, period, and their interaction as fixed effects to examine their effects on behavioral responses. A Gamma Regression with a log-link function was used for latencies to enter and bleat, which represent continuous duration data; this included the same independent variables (treatment, period, and interaction). Post-hoc tests were conducted using Bonferroni correction to compare pairwise differences between feed types. *p* < 0.05 was considered statistically significant.

Two-way analysis of variance was performed to confirm the effects of treatment and period on ruminal VFA, ruminal and plasma ammonia, plasma cortisol, and aldosterone concentrations. The model included the treatment, period, and treatment-by-period interactions as factors. Post-hoc tests were conducted using Tukey’s HSD to compare pairwise differences between feeding treatments; *p* < 0.05 was considered statistically significant. We performed Spearman correlation analysis to examine the relationships among VFAs, rumen ammonia, plasma ammonia, plasma hormones, and behavioral variables in the OFT. All statistical analyses were performed using SPSS (16).

Baseline characteristics (body weight and age) were compared among the three treatment groups using the Kruskal–Wallis test, and sex ratios were compared using Fisher’s exact test, to confirm that groups were balanced prior to the experiment.

## 3 Results

### 3.1 Behavioral stress responses in the OFT

**Figure 2:**
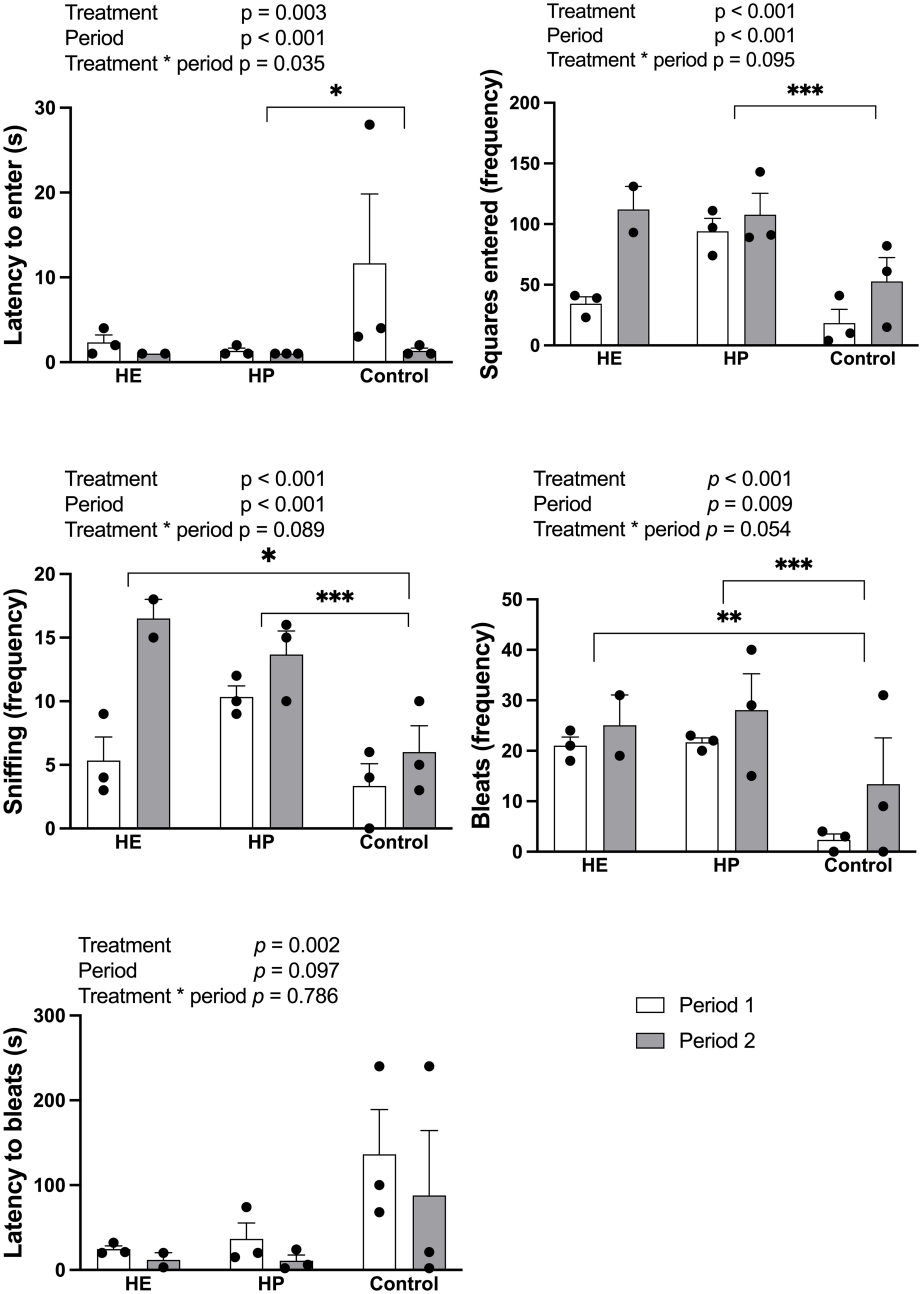
Effect of different energy level and protein level of feed on the behaviors measured in the open field test, 2 h post-feeding. HE represents high energy group; HP represents high protein group. Data are expressed as the mean and standard error, SE, with individual data points overlaid on the bars. Open bars represent period 1, and filled bars represent period 2. Asterisks indicate statistically significant pairwise differences between feed groups; *p < 0.05; **p < 0.01; ***p < 0.001. Generalized Linear Model with Negative Binomial Regression (for frequency data) and Gamma Regression (for duration data), both with a log link function; Bonferroni-adjusted (*n* = 5 for HE, *n* = 6 for HP and control).

Treatment had a significant effect on latency to enter (Wald χ^2^ = 11.87, df = 2, *p* = 0.003). Sheep in the control group took longer to enter the open field pen (6.5 ± 4.3 s) than those in the HP group (1.2 ± 0.2 s; *p* < 0.05), whereas the HE (1.8 ± 0.6 s) group did not significantly differ from the other groups (Figure 2). The frequency of squares entered also showed a significant treatment effect (Wald χ^2^ = 20.20, df = 2, *p* < 0.001); the HP group (100.8 ± 9.8 squares) entered more squares than in the control group (35.5 ± 12.8 squares) (*p* < 0.001). The HE group entered 65.4 ± 20.2 squares, which was not significantly different from the control group (*p* = 0.080). Sniffing was significantly affected by treatment (Wald χ^2^ = 18.02, df = 2, *p* < 0.001); it was more frequently observed in the HP (12.0 ± 1.2 times; *p* < 0.001) and HE (9.8 ± 3.0 times, *p* = 0.010) groups than in the control group (4.7 ± 1.4 times). Bleat frequency also differed among groups (Wald χ^2^ = 21.19, df = 2, *p* < 0.001); it was significantly higher in the HP (22.6 ± 2.3 times) (*p* < 0.001) and HE (24.8 ± 3.6 times) (*p* = 0.005) groups than in the control group (7.8 ± 4.8 times). The latency to bleat was 23.5 ± 10.7, 19.2 ± 4.6, and 111.8 ± 42.9 s in the HP, HE, and control groups, respectively. Although these were statistically significant treatment effects (Wald χ^2^ = 12.67, df = 2, *p* = 0.002), post-hoc pairwise comparisons revealed no significant differences between each group after Bonferroni correction. In the OFT, significant differences in latency to enter (Wald χ^2^ = 12.18, df = 1, *p* < 0.001), squares entered (Wald χ^2^ = 12.79, df = 1, *p* < 0.001), sniffing (Wald χ^2^ = 13.07, df = 1, *p* < 0.001), and bleats were observed between the two periods (Wald χ^2^ = 6.92, df = 1, *p* = 0.009).

### 3.2 Hormone concentrations in the blood

**Figure 3:**
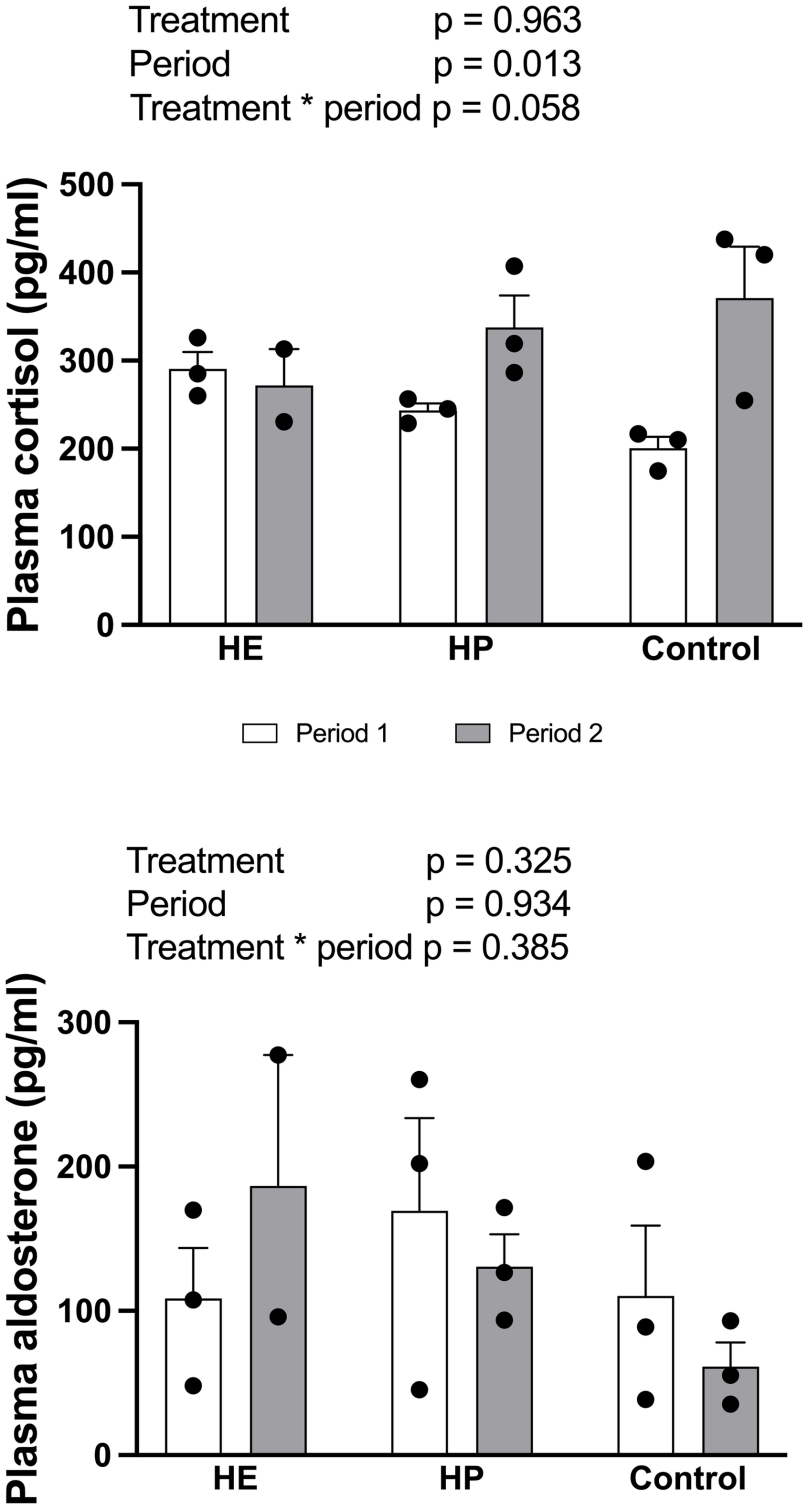
Effect of different energy level and protein level of feed on the plasma concentration of cortisol and aldosterone 2 h post-feeding. HE represents high energy group; HP represents high protein group. Data are expressed as the mean and standard error, SE, with individual data points overlaid on the bars. Open bars represent period 1, and filled bars represent period 2. Asterisks indicate statistically significant pairwise differences between feed groups (*p < 0.05, **p < 0.01; ***p < 0.001; two-way ANOVA with Tukey’s HSD *post-hoc* test; *n* = 5 for HE, *n* = 6 for HP and control).

Plasma cortisol levels did not differ between the feeding treatments [F (2, 11) = 0.04, *p* = 0.963] (Figure 3). A period effect was detected [F (1, 11) = 8.71, *p* = 0.013]; higher values were observed in Period 2 than in Period 1. Similarly, plasma aldosterone levels showed no significant differences between feed types or periods [F (2, 11) = 1.25, *p* = 0.325 for treatment; F (1, 11) = 0.01, *p* = 0.934 for period].

### 3.3 Ruminal VFA concentrations

**Figure 4:**
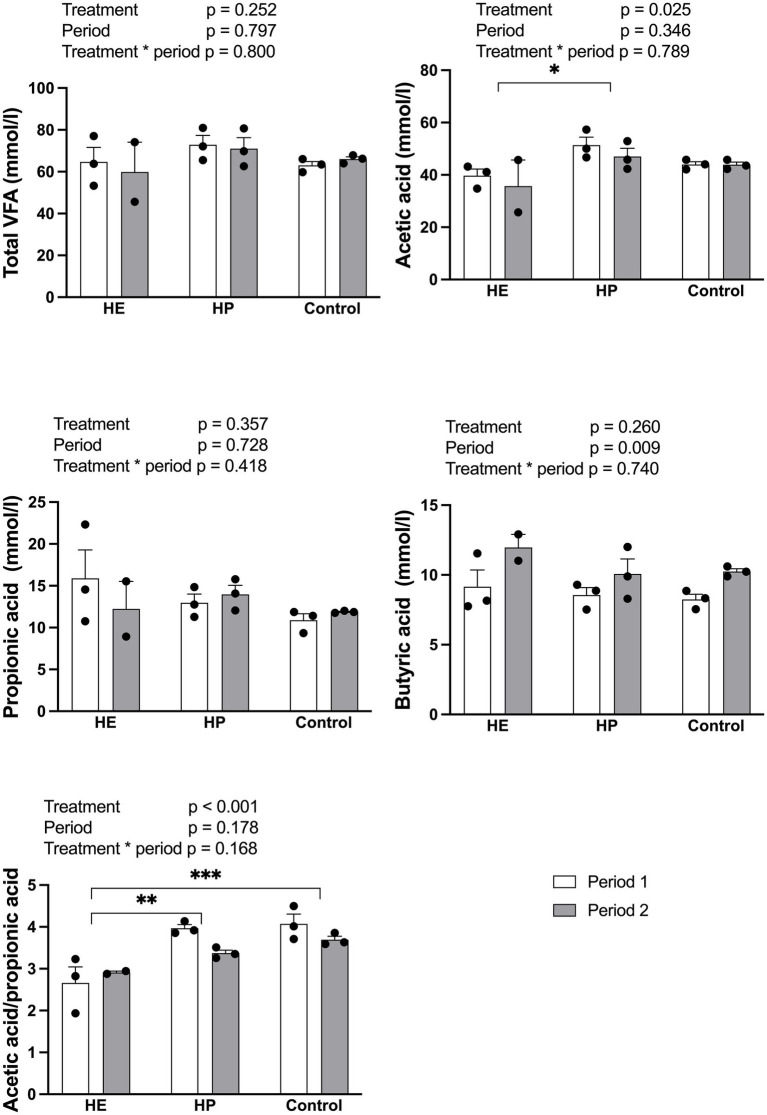
Effect of different energy level and protein level of feed on the rumen VFA concentration 2 h post-feeding. HE represents high energy group; HP represents high protein group. Data are expressed as the mean and standard error, SE, with individual data points overlaid on the bars. Open bars represent period 1, and filled bars represent period 2. Asterisks indicate statistically significant pairwise differences between feed groups (*p < 0.05; **p < 0.01; ***p < 0.001; two-way ANOVA with Tukey’s HSD *post-hoc* test; *n* = 5 for HE, *n* = 6 for HP and control).

A significant difference in the concentration of acetic acid and A/P ratio was observed between the feeding treatments (Figure 4). Acetic acid showed a treatment effect [F (2, 11) = 5.24, *p* = 0.025] driven by lower concentrations in the HE group (38.10 ± 3.51 mM) than in the HP group (49.17 ± 2.20 mM; *p* = 0.027), with no difference compared with the control group (43.92 ± 0.66 mM). The A/P ratio was significantly affected by treatment [F (2, 11) = 14.64, *p* < 0.001] and was lower in the HE group than in the control and HP groups (HE 2.76 ± 0.22 vs. control 3.88 ± 0.14, *p* = 0.001; vs. HP 3.67 ± 0.14, *p* = 0.003). Propionic acid content did not differ according to feeding treatment [F (2, 11) = 1.13, *p* = 0.357]. Only butyric acid showed a period effect [F (1, 11) = 10.02, *p* = 0.009]. No significant effect on the total rumen VFA concentration was observed [F (2, 11) = 1.57, *p* = 0.252].

### 3.4 Ruminal ammonia concentrations

**Figure 5:**
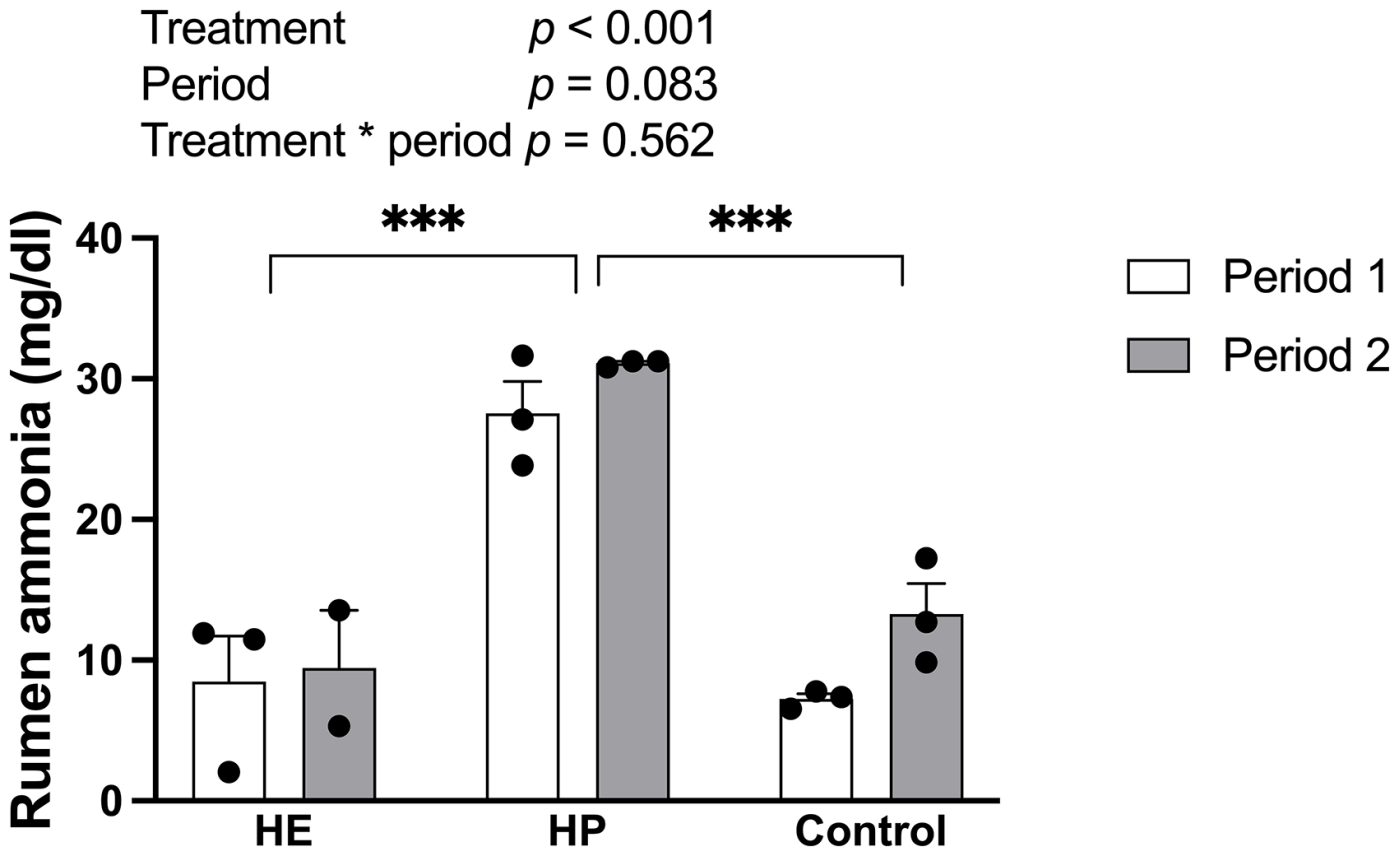
Effect of different energy level and protein level of feed on the rumen ammonia concentration 2 h post-feeding. HE represents high energy group; HP represents high protein group. Data are expressed as the mean and standard error, SE, with individual data points overlaid on the bars. Open bars represent period 1, and filled bars represent period 2. Asterisks indicate statistically significant pairwise differences between feed groups (*p < 0.05; **p < 0.01; ***p < 0.001; two-way ANOVA with Tukey’s HSD post-hoc test; *n* = 5 for HE, *n* = 6 for HP and control).

The feeding treatment had a significant effect on rumen ammonia concentration [F (2, 11) = 52.68, *p* < 0.001] (Figure 5). Bonferroni post-hoc comparisons showed that rumen ammonia levels were significantly higher in the HP group (29.32 ± 1.29 mg/dL) than in both the control (10.27 ± 1.67 mg/dL) (*p* < 0.001) and HE (8.87 ± 2.20 mg/dL) (*p* < 0.001) groups.

### 3.5 Plasma ammonia concentrations

**Figure 6:**
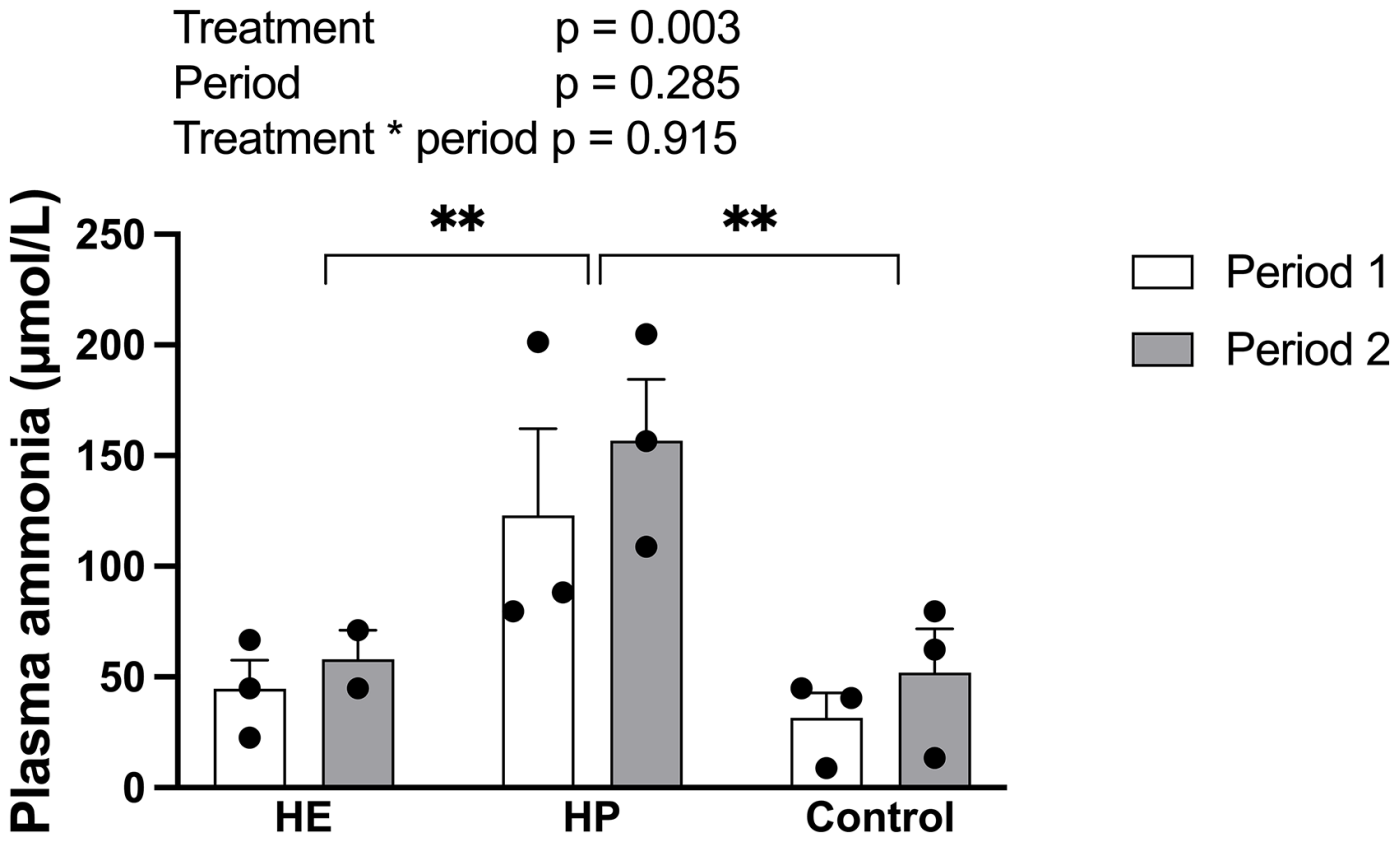
Effect of different energy level and protein level of feed on the plasma concentration of ammonia 2 h post-feeding. HE represents high energy group; HP represents high protein group. Data are expressed as the mean and standard error, SE, with individual data points overlaid on the bars. Open bars represent period 1, and filled bars represent period 2. Asterisks indicate statistically significant pairwise differences between feed groups (*p < 0.05; ** *p* < 0.01; ***p < 0.001; two-way ANOVA with Tukey’s HSD post-hoc test; *n* = 5 for HE, *n* = 6 for HP and control).

The plasma ammonia concentration differed significantly among treatments [F (2, 11) = 10.31, *p* = 0.003] and was significantly higher in the HP group than in the HE (*p* = 0.012) and control (*p* = 0.005) groups (Figure 6). There were no significant effects of period [F (1, 11) = 1.26, *p* = 0.285] or the interaction between treatment and period [F (2, 11) = 0.09, *p* = 0.915].

### 3.6 Relationship between ruminal fermentation products, blood ammonia, plasma hormones and behavioral stress responses

For the VFAs, a positive trend was observed between rumen propionic acid and sniffing (*ρ* = 0.413, *p* = 0.100) (Table 4). Furthermore, rumen propionic acid was positively correlated with plasma cortisol concentration (*ρ* = 0.512, *p* = 0.036). Rumen butyric acid showed a tendency toward a negative correlation with latencies to enter (*ρ* = −0.446, *p* = 0.073) and bleat (*ρ* = −0.417, *p* = 0.096). Butyric acid levels were significantly and positively correlated with plasma cortisol levels (*ρ* = 0.485, *p* = 0.048). The A/P ratio tended to be positively correlated with latency to bleat (*ρ* = 0.437, *p* = 0.079) and negatively correlated with plasma cortisol concentration (*ρ* = −0.561, *p* = 0.019).

**Table 4:** Spearman’s correlation analysis of ruminal fermentation products, blood biomarkers, and behavioral stress responses.

Variables	Volatile fatty acids					Ammonia		Plasma hormones	
Open-field behaviors	Acetic acid	Propionic acid	Butyric acid	Total VFA	Acetic acid/	Rumen ammonia			
					Propionic acid		Plasma ammonia	Cortisol	Aldosterone
Latency to enter	−0.112	−0.200	−0.446^†^	−0.208	0.275	−0.536^*^	−0.695^**^	−0.574^*^	−0.320
Squares entered	0.213	0.125	0.161	0.116	−0.146	0.534^*^	0.777^***^	0.266	0.313
Sniffing	0.344	0.413	0.404	0.342	−0.265	0.455^†^	0.569^*^	0.254	0.424^†^
Latency to bleat	−0.113	−0.246	−0.417^†^	−0.247	0.437^†^	−0.276	−0.517^*^	−0.642^**^	0.092
Bleats	−0.036	0.164	0.185	0.063	−0.412	0.298	0.540^*^	0.483^*^	0.261
Plasma hormones									
Cortisol	−0.012	0.512^*^	0.485^*^	0.350	−0.561^*^	0.341	0.440^†^		
Aldosterone	0.319	0.360	0.255	0.380	−0.140	0.394	0.404		

Variables are pooled across treatments and periods (*n* = 17). *p < 0.05; **p < 0.01; ***p < 0.001; ^†^0.05 ≤ *p* < 0.10.

Ruminal and plasma ammonia concentrations were strongly associated with OFT behavior variables. Ruminal ammonia levels were negatively correlated with latency to enter (*ρ* = −0.536, *p* = 0.027) and positively correlated with the frequency of squares entered (*ρ* = 0.534, *p* = 0.027). A positive correlation was also observed between ruminal ammonia concentrations and sniffing (*ρ* = 0.455, *p* = 0.066). The plasma ammonia levels showed a stronger pattern. Plasma ammonia concentration was associated with shorter latency to enter (*ρ* = −0.695, *p* = 0.002), greater frequency of squares entered (*ρ* = 0.777, *p* < 0.001), more sniffing (*ρ* = 0.569, *p* = 0.017), more bleats (*ρ* = 0.540, *p* = 0.025), and shorter latency to bleat (*ρ* = −0.517, *p* = 0.034). A positive association with plasma cortisol levels was also observed (*ρ* = 0.440, *p* = 0.077).

Plasma cortisol concentration negatively correlated with latency to enter (*ρ* = −0.574, *p* = 0.016) and latency to bleat (*ρ* = −0.642, *p* = 0.005). In contrast, it was positively associated with bleats (*ρ* = 0.483, *p* = 0.049).

## 4 Discussion

This study showed that feed composition, particularly dietary protein level, was associated with altered behavioral stress responses in sheep. Sheep fed the HP diet exhibited markedly increased locomotion and bleating during the OFT. These behavioral variables are commonly used as indicators of behavioral stress responses (1, 2). However, these OFT behaviors may also reflect stress-related responsiveness, arousal, exploratory drive, or sensitivity to social isolation, rather than negative affect alone (1–3). The latency to enter the field was significantly shorter in the HP group, and although the overall treatment effect on latency to bleat was significant, post-hoc pairwise comparisons did not reach statistical significance after Bonferroni correction. Nevertheless, the numerical trend toward shorter latency to bleat in the HP and HE groups is consistent with the pattern observed for the other behavioral variables. The behavioral responses observed in the HP group may be associated with elevated ruminal and plasma ammonia concentrations. High dietary protein or concentrate levels can significantly alter ruminal fermentation profiles and systemic nitrogen metabolism (17, 18). The HP diet contained 18.9% DM crude protein, approximately twice that of the control (9.5% DM), with soybean meal as the primary protein source. Soybean meal protein is highly rumen-degradable, and the relatively low energy content of the HP diet (TDN 63.1% DM) likely created an imbalance between nitrogen release and energy availability for microbial protein synthesis, resulting in excess ammonia accumulation in the rumen. Excess ruminal ammonia may be absorbed into the blood, and elevated circulating ammonia can cross the blood–brain barrier and disrupt neurotransmission through mechanisms involving astrocyte swelling, glutamate-gamma-aminobutyric acid (GABA) imbalance, and oxidative stress. Evidence from mammalian experimental models indicates that hyperammonemia can alter behavior and neural function (11, 19–22). Therefore, the elevated plasma ammonia observed in the HP group may have contributed to the altered behavioral responses seen in the OFT, although the causal pathway remains to be confirmed in sheep. These neurobehavioral alterations are consistent with findings in rodent models, where chronic moderate hyperammonemia impaired avoidance behavior and modulated locomotor activity by activating NMDA-type glutamate receptors (15, 23). Furthermore, elevated ammonia levels disturb the balance between glutamate and GABA, leading to stress-related behaviors in the OFT (22). These findings are consistent with the hypothesis that high protein intake elevates circulating ammonia levels, which may be associated with enhanced stress-related behavioral responses in sheep. Although interventional studies (e.g., ammonia modulation or neurotransmission antagonism) are required for causal validation, the present findings align with the established neurobiology of ammonia toxicity. The positive correlation between the plasma ammonia and cortisol levels is consistent with this interpretation. However, because the OFT was conducted on day 1 and rumen fluid was collected on day 4 of each sampling phase, the correlations between rumen parameters and behavioral variables should be interpreted as reflecting feed-induced steady-state differences rather than acute within-day effects.

The HE feed altered ruminal fermentation profiles, which were characterized by a significant decrease in acetic acid and a reduced A/P ratio. These results are consistent with the known metabolic shifts associated with high-starch diets (24, 25). The HE group showed a higher frequency of sniffing and bleats than the control group. These behavioral findings are consistent with those of our previous study (9), wherein HE feed increased fear reactivity in sheep. Lower and higher proportions of acetic and butyric acids were observed, respectively. In the same study, the A/P ratio showed a positive correlation with latency to bleat and was negatively correlated with plasma cortisol concentrations, suggesting a link between ruminal fermentation profiles and stress responses in sheep (9). For example, vocalizations maintain contact with conspecifics and are often used as indicators of fear responses (2, 3, 26). In the present study, the latency to bleat was negatively associated with plasma cortisol levels. This may have been caused by changes in the ruminal A/P ratio. However, cortisol levels did not differ significantly between the feeding treatments. Therefore, further investigation is required to determine the relationship between the rumen A/P ratio, cortisol levels, and stress responsiveness. An alternative explanation for the behavioral changes observed in the HE group is subacute ruminal acidosis (SARA), which can occur with high-concentrate diets and may cause gastrointestinal discomfort that alters behavior (27, 28). However, several observations argue against this possibility in the present study. The total dry matter offered was 2.4% of body weight, only approximately 20% above maintenance requirements, which substantially limits the absolute amount of concentrate ingested. Commun et al. (28) reported that the risk of SARA in sheep fed a 60:40 concentrate-to-forage ratio was primarily associated with the total amount of feed ingested under ad libitum conditions, rather than the concentrate ratio alone. In the present study, no clinical signs of acidosis, such as reduced feed intake, diarrhea, or lethargy, were observed during daily health monitoring, and orts remained below 5% of the offered amount throughout the experiment. Furthermore, total VFA concentrations did not differ significantly among the three groups, suggesting that overall fermentation intensity was not abnormally elevated. Nevertheless, ruminal pH was not measured in this study, and future studies should include ruminal pH monitoring to evaluate the potential contribution of acidosis to behavioral responses.

Plasma cortisol levels showed a significant period effect; higher levels were observed during Period 2 (June 2019). According to the Japan Meteorological Agency (Kawatabi AMeDAS station, Osaki, Miyagi), the mean ambient temperature during the sampling period was −1.8 °C for Period 1 (January 25–28, 2019) and 18.0 °C for Period 2 (June 27–30, 2019), indicating a substantial seasonal difference between the two periods (29, 30). Maurya et al. (31) reported that Malpura sheep exposed to heat stress at 35–41 °C showed significantly elevated cortisol levels. Although the ambient temperature during Period 2 in the present study (18.0 °C) was well below such heat stress thresholds, the temperature difference between the two periods (~20 °C), together with differences in animal age and body weight, may have contributed to the observed period effect on cortisol (29). On the other hand, the effects of feeding treatments on plasma cortisol concentrations were not significant. Nevertheless, correlation analyses indicated an association between rumen fermentation indicators and plasma cortisol concentrations. Plasma aldosterone levels did not differ significantly across treatments or periods, indicating that aldosterone may not play a central role in mediating feed–stress effects under the conditions tested. Nonetheless, the consistent effect of the HP feed on behavior across both periods indicates that feed composition affects behavioral stress responses regardless of season.

This study has several limitations that should be considered when interpreting the results. The sample size was small (*n* = 5–6 per group), which limits statistical power and generalizability. This may partly explain why some variables, such as plasma cortisol and aldosterone, did not show significant treatment effects. In addition, ruminal pH was not measured, preventing a direct evaluation of the potential role of subacute ruminal acidosis. Future studies with larger sample sizes and continuous ruminal pH monitoring are needed to confirm and extend these findings.

In conclusion, this study not only verifies our prior observation that rumen VFA profiles relate to behavioral stress responses but also provides evidence that HP feed increases ruminal and plasma ammonia levels and is associated with more stress-related behavioral responses in ruminants. These results highlight the importance of considering stress responses, particularly behavioral variables, when formulating ruminant feed. Furthermore, they support the use of behavioral tests as practical tools in feed–stress research and livestock welfare assessment. These findings underscore the importance of well-balanced feed protein in ruminant nutrition. Although protein is necessary for productivity, excess levels may elevate blood ammonia concentrations and trigger stress-related behavioral changes. Thus, this study provides preliminary evidence supporting a link between rumen metabolic changes and behavioral stress responses in sheep. Future studies should explore the neuroendocrine pathways linking feed components to behavior, including biomarkers other than cortisol and aldosterone.

## Data Availability

The original contributions presented in the study are included in the article/supplementary material, further inquiries can be directed to the corresponding author.
